# Generalizability of FIGARO‐DKD and FIDELIO‐DKD Trial Criteria to the US Population Eligible for Finerenone

**DOI:** 10.1161/JAHA.121.025079

**Published:** 2022-03-21

**Authors:** Nicholas Chiu, Rahul Aggarwal, George L. Bakris, Bertram Pitt, Deepak L. Bhatt

**Affiliations:** ^1^ Department of Medicine Beth Israel Deaconess Medical Center Harvard Medical School Boston MA; ^2^ University of Chicago IL; ^3^ University of Michigan Ann Arbor MI; ^4^ Brigham and Women’s Hospital Heart and Vascular Center Harvard Medical School Boston MA

**Keywords:** albuminuria, CKD, diabetes, finerenone, generalizability, Cardiovascular Disease, Diabetes, Type 2, Epidemiology, Nephrology and Kidney, Quality and Outcomes

The FIDELIO‐DKD (Finerenone in Reducing Kidney Failure and Disease Progression in Diabetic Kidney Disease)^1^ and the FIGARO‐DKD (Finerenone in Reducing Cardiovascular Mortality and Morbidity in Diabetic Kidney Disease)^2^ trials demonstrated benefits for finerenone in reducing risk for kidney and cardiovascular outcomes in patients with type 2 diabetes and chronic kidney disease (CKD). FIDELIO‐DKD and FIGARO‐DKD enrolled patients with different levels of kidney dysfunction.

With established benefits, finerenone could improve outcomes in many patients with type 2 diabetes and CKD. However, this population has faced challenges in uptake of guideline‐directed therapy. Thus, determining the number of eligible US individuals can serve as a benchmark for national implementation success. We apply FIDELIO‐DKD and FIGARO‐DKD enrollment criteria to the US population, assess characteristics of the eligible population, and determine the number of individuals eligible for finerenone.

All data used are publicly available, and the data and analytical code that support the findings of this study are available from the corresponding author upon reasonable request. FIDELIO‐DKD and FIGARO‐DKD enrollment criteria were applied to the publicly available National Health and Nutrition Examination Survey data sets (NHANES, 2009–2018). The NHANES survey is designed to represent the US population by using complex, multistage, stratified, clustered samples of the civilian noninstitutionalized populations. From this, sample weights are used to yield nationally representative estimates. In this study, individuals who were ≥18 years of age, had type 2 diabetes, had serum potassium ≤4.8 mmol/L, and were on angiotensin‐converting enzyme inhibitor or angiotensin receptor blocker therapy were included. Patients with heart failure were excluded. In the FIGARO‐DKD cohort, inclusion criteria included a urinary albumin‐to‐creatinine ratio (UACR) of ≥30 and <300 mg/g with an estimated glomerular filtration rate (eGFR) of ≥25 and ≤90 mL/min per 1.73 m^2^ or included a UACR of ≥300 and ≤5000 mg/g with an estimated glomerular filtration rate ≥60 mL/min per 1.73 m^2^. The FIDELIO‐DKD inclusion criteria were a UACR ≥30 of <300 mg/g with an estimated glomerular filtration rate ≥25 but <60 mL/min per 1.73 m² or a UACR ≥300 and ≤5000 mg/g with an estimated glomerular filtration rate ≥25 to <75 mL/min per 1.73 m². We further derived a combined cohort of patients meeting at least 1 trial criterion and described baseline characteristics. Analyses were conducted with R 4.0.5 and survey weights were used to determine national projections. NHANES was approved by the National Center for Health Statistics Institutional Review Board. As part of the NHANES data collection, a consent form was signed by all participants in the survey.

FIDELIO‐DKD trial criteria applied to 1 022 705 (95% CI, 830 876–1 214 533) individuals in the United States, and FIGARO‐DKD trial criteria applied to 1 980 176 (95% CI, 1 706 544–2 253 807) individuals. A total of 2 232 031 (95% CI, 1 947 816–2 516 246) individuals in the United States met criteria for initiation of finerenone by at least 1 full trial criterion. Compared with FIDELIO‐DKD trial participants, the corresponding eligible US population had a higher proportion of women (45.1% [95% CI, 36.6–53.5%] versus 31.3%) and non‐Hispanic Black adults (14.5% [95% CI, 9.7–19.2%] versus 4.9%). The US population had different albuminuria profiles: both FIDELIO‐DKD and FIGARO‐DKD eligible US populations had lower median UACRs at 144.3 mg/g (87.1–309.4) and 93.6 mg/g (77.9–119.0), respectively, compared with 833 mg/g (441–1628) and 302 mg/g (105–749) for trial participants. Characteristics of trial populations and US population meeting eligibility criteria are displayed in Table.

Approximately 1 million US individuals were eligible for treatment under FIDELIO‐DKD criteria, which included predominantly stage 3 or 4 CKD. In contrast, almost 2 million US individuals qualified for treatment under FIGARO‐DKD criteria, which expanded inclusion to stage 1 or 2 CKD with severely elevated albuminuria and stage 2 to 4 CKD with moderately elevated albuminuria. Overall, 2.2 million individuals in the United States would qualify for finerenone by at least 1 full trial criterion.

Though age, body mass index, glycated hemoglobin, and other comorbidities were similar between trial participants, key differences were noted regarding kidney disease severity. First, the US population has fewer individuals with severe albuminuria (>300 mg/g), and more individuals with moderately elevated albuminuria (30–300 mg/g). Notably, in the FIDELIO‐DKD trial, over 87% of patients had severe albuminuria. In contrast, only 40% of the FIDELIO trial‐eligible US population had this degree of albuminuria. FIGARO‐DKD trial showed the benefits of finerenone extend to patients with less severe albuminuria, because 46.8% of patients had moderate albuminuria. Notably, the US population had a substantially higher rate of moderate albuminuria, at 78.5% of FIGARO‐DKD eligible US individuals versus 46.8% in the trial. Moderate albuminuria marks early CKD and treating CKD earlier may slow disease progression. Microalbuminuria screening is cost effective in patients with diabetes,^3^ and improved CKD screening practices could identify individuals likely to benefit from finerenone. Further studies should examine the effect of concurrent use of sodium‐glucose cotransporter‐2 inhibitors, as this medication is indicated for many individuals with diabetes and CKD,^4^ and understanding potential crowd‐out of other therapies is important. A limitation is that we did not factor in cost, which may limit population uptake. Further, whereas both trials excluded only heart failure with reduced ejection fraction patients, we excluded patients with heart failure irrespective of ejection fraction because NHANES does not include ejection fraction data. Nevertheless, this would only underestimate the number of individuals eligible for finerenone. In conclusion, FIDELIO‐DKD and FIGARO‐DKD are broadly generalizable to the US population with CKD and diabetes and apply to over 2 million eligible US adults. Strategies to address access and expand uptake are needed.

## Sources of Funding

None.

## Disclosures

Dr. George L. Bakris discloses the following relationships: Consultant: Merck, Bayer, Vascular Dynamics, KBP Biosciences, Ionis, Alnylam, Astra Zeneca, Quantum Genomics, Horizon, Novo Nordisk; Research support: Steering committee of trials ‐ Bayer, Vascular Dynamics, Quantum Genomics, Alnylam, Novo Nordisk; Editor: *American Journal of Nephrology*. Dr. Bertram Pitt discloses the following relationships: consulting fees from Bayer, Astra Zeneca, Boehringer Ingelheim/Lilly, and Phasebio; consulting fees and stock options from SCPharmaceuticals, SQinnovations, G3pharmaceuticals, Relypsa/Vifor, Cereno scientific, KBP Pharmaceuticals, Sarfez, Tricida, Proton Intel, and Brainstorm Medical. Dr. Pitt is chairman of the steering committee for the National Heart, Lung, and Blood Institute’s TRANSFORM (Torsemide Comparison With Furosemide For Management of Heart Failure) trial and co‐chair of SPIRRIT ([Spironolactone Initiation Registry Randomized Interventional Trial] from the National Heart, Lung, and Blood Institute–Swedish Heart Foundation). He holds US Patent No. 9931412 on site‐specific delivery of eplerenone to the myocardium and has a pending US Patent (63/045,784) on histone acetylation–modulating agents for the treatment and protection of organ damage. Dr. Deepak L. Bhatt discloses the following relationships ‐ Advisory Board: Bayer, Boehringer Ingelheim, Cardax, CellProthera, Cereno Scientific, Elsevier Practice Update Cardiology, Janssen, Level Ex, Medscape Cardiology, MyoKardia, NirvaMed, Novo Nordisk, PhaseBio, PLx Pharma, Regado Biosciences, Stasys; Board of Directors: Boston VA Research Institute, Society of Cardiovascular Patient Care, TobeSoft; Chair: Inaugural Chair, American Heart Association Quality Oversight Committee; Data Monitoring Committees: Baim Institute for Clinical Research (formerly Harvard Clinical Research Institute, for the PORTICO trial, funded by St. Jude Medical, now Abbott), Boston Scientific (Chair, PEITHO trial), Cleveland Clinic (including for the ExCEED trial, funded by Edwards), Contego Medical (Chair, PERFORMANCE 2), Duke Clinical Research Institute, Mayo Clinic, Mount Sinai School of Medicine (for the ENVISAGE trial, funded by Daiichi Sankyo), Novartis, Population Health Research Institute; Honoraria: American College of Cardiology (Senior Associate Editor, Clinical Trials and News, ACC.org; Chair, ACC Accreditation Oversight Committee), Arnold and Porter law firm (work related to Sanofi/Bristol‐Myers Squibb clopidogrel litigation), Baim Institute for Clinical Research (formerly Harvard Clinical Research Institute; RE‐DUAL PCI clinical trial steering committee funded by Boehringer Ingelheim; AEGIS‐II executive committee funded by CSL Behring), Belvoir Publications (Editor in Chief, *Harvard Heart Letter*), Canadian Medical and Surgical Knowledge Translation Research Group (clinical trial steering committees), Cowen and Company, Duke Clinical Research Institute (clinical trial steering committees, including for the PRONOUNCE trial, funded by Ferring Pharmaceuticals), HMP Global (Editor in Chief, *Journal of Invasive Cardiology*), *Journal of the American College of Cardiology* (Guest Editor; Associate Editor), K2P (Co‐Chair, interdisciplinary curriculum), Level Ex, Medtelligence/ReachMD (continuing medical education steering committees), MJH Life Sciences, Piper Sandler, Population Health Research Institute (for the COMPASS operations committee, publications committee, steering committee, and USA national co‐leader, funded by Bayer), Slack Publications (Chief Medical Editor, *Cardiology Today’s Intervention*), Society of Cardiovascular Patient Care (Secretary/Treasurer), WebMD (continuing medical education steering committees); Other: *Clinical Cardiology* (Deputy Editor), NCDR‐ACTION Registry Steering Committee (Chair), VA CART Research and Publications Committee (Chair); Research Funding: Abbott, Afimmune, Amarin, Amgen, AstraZeneca, Bayer, Boehringer Ingelheim, Bristol‐Myers Squibb, Cardax, CellProthera, Cereno Scientific, Chiesi, CSL Behring, Eisai, Ethicon, Faraday Pharmaceuticals, Ferring Pharmaceuticals, Forest Laboratories, Fractyl, Garmin, HLS Therapeutics, Idorsia, Ironwood, Ischemix, Janssen, Javelin, Lexicon, Lilly, Medtronic, MyoKardia, NirvaMed, Novartis, Novo Nordisk, Owkin, Pfizer, PhaseBio, PLx Pharma, Regeneron, Reid Hoffman Foundation, Roche, Sanofi, Stasys, Synaptic, The Medicines Company, 89Bio; Royalties: Elsevier (Editor, *Cardiovascular Intervention: A Companion to Braunwald’s Heart Disease*); Site Co‐Investigator: Abbott, Biotronik, Boston Scientific, CSI, St. Jude Medical (now Abbott), Philips, Svelte; Trustee: American College of Cardiology; Unfunded Research: FlowCo, Merck, Takeda. The remaining authors have no disclosures to report.

**Table 1:** Comparison of FIGARO and FIDELIO Trial Populations With US Population Meeting Trial Eligibility Criteria

Characteristics	FIDELIO trial	FIGARO trial	United States (FIDELIO)	United States (FIGARO)	United States (combined)
	n=5734	n=7437	n=1 022 705 (±97 874)	n=1 980 176 (±139 610)	n=2 232 031 (±145 010)
Age, mean, y	65.4 (±8.9)	64.1 (±9.7)	68.6 (±1.2)	67.0 (±0.7)	67.0 (±0.03)
Sex (%)					
Male	68.9%	68.6%	54.9% (±4.3%)	60.0% (±3.4%)	58.5% (±3.1%)
Female	31.3%	31.4%	45.1% (±4.3%)	40.0% (±3.4%)	41.5% (±3.1%)
Race (%)					
Non‐Hispanic White	62.7%	72.5%	59.6% (±4.2%)	58.0% (±3.0%)	57.9% (±3.0%)
Non‐Hispanic Black	4.9%	3.1%	14.4% (±2.4%)	16.2% (±2.1%)	16.3% (±1.9%)
Other	32.3%	24.2%	26.0% (±4.1%)	25.8% (±3.1%)	25.8% (±3.0%)
Blood pressure, mean, mm Hg					
Systolic	138.1 (±14.3)	135.8 (±14.0)	138.4 (±2.6)	136.9 (±1.9)	138.2 (±1.7)
Diastolic	75.8 (±9.7)	76.8 (±9.5)	67.0 (±1.3)	68.2 (±1.3)	68.5 (±1.2)
Body mass index, mean, kg/m^2^	31.1 (±6.0)	31.5 (±6.0)	32.3 (±0.7)	32.0 (±0.5)	32.2 (±0.4)
Hemoglobin A1c mean, %	7.7 (±1.3)	7.7 (±1.4)	7.5 (±0.1)	7.6 (±0.1)	7.6 (±0.1)
Serum creatinine mean, mg/dL			1.4 (±0.02)	1.1 (±0.02)	1.1 (±0.02)
Estimated glomerular filtration rate, mean, mL/min per 1.73 m^2^	44.4 (±12.5)	67.6 (±21.7)	49.4 (±1.2)	68.3 (±1.4)	65.7 (±1.4)
≥60	11.2%	62.0%	15.4% (±4.3%)	69.0% (±3.7%)	61.2% (±3.7%)
45–59	34.3%	20.2%	49.7% (±5.9%)	19.1% (±3.6%)	22.7% (±3.7%)
25–44	52.1%	17.4%	35.0% (±4.4%)	11.8% (±1.6%)	16.0% (±2.0%)
<25	2.3%	0.4%	0	0	0
Urine albumin‐creatinine ratio, median, mg/g	833 (441–1628)	302 (105–749)	144.3 (87.1–309.4)	93.6 (77.9–119.0)	110.2 (91.1–135.0)
30–300 mg/g	12.4%	46.8%	60.0% (±5.1%)	78.5% (±3.4%)	69.7% (±3.5%)
>300 mg/g	87.2%	50.2%	40.0% (±5.1%)	21.4% (±3.4%)	30.3% (±3.5%)
Serum potassium, mean, mmol/L	4.37±0.46	4.33±0.43	4.2 (±0.04)	4.1 (±0.02)	4.1 (±0.02)
Myocardial infarction, %	13.3%	7.4%	13.1% (±2.7%)	10.8% (±2.1%)	10.9% (±1.9%)
Stroke, %	11.6%	12.0%	14.9% (±3.2%)	11.0% (±2.1%)	11.7% (±2.1%)

Demographics of patients in FIGARO and FIDELIO Trials and demographics of US population meeting trial eligibility criteria. All national estimates were projections for the United States accounting for National Health and Nutrition Examination Survey sample weights. FIDELIO‐DKD indicates Finerenone in Reducing Kidney Failure and Disease Progression in Diabetic Kidney Disease; and FIGARO‐DKD, Finerenone in Reducing Cardiovascular Mortality and Morbidity in Diabetic Kidney Disease.

The term "Other" refers to "Mexican American", "Other Hispanic", and "Other Race ‐ Including Multi‐Racial" as designated in NHANES.
